# Poisoning with Morphine

**Published:** 1866-12

**Authors:** J. L. Prentiss

**Affiliations:** Lawrence, Kansas


*Poisoning with Morphine.* Reported by J. L. Prentiss, M.D., of Lawrence, Kansas.

Miss L. S——, aged 25 years, swallowed five grains of morphine at 5 o'clock P. M. on the 28th of August. At 11 P. M. I was called, and, on entering the room, found her in a state of complete insensibility. Pulse beat 80 per minute, moderately full; the pupils were contracted; breathing stertorous, and at times ceased entirely for the space of a minute; the skin was cold and clammy, and narcotism was so complete, that it seemed impossible to arouse her sufficiently to administer an antidote, as the masseter muscles were rigid, and the teeth so firmly closed, that it was impossible to open the mouth.

By the application of cold to the head, and cold douche to the face and chest, besides anything but a moderate amount of shaking, we succeeded, after about half an hour, in getting her sufficiently aroused to take medicine. I immediately gave her one drachm of the tincture of belladonna, and repeated the dose every half hour for two hours, when the pupils became dilated, the breathing more natural, and the patient seemed comparatively free from the influence of the narcotic.

In this case I used nothing but the belladonna and strong coffee, and am well satisfied that the former should be classed among the first as an antidote in cases of poisoning by opium.

